# Complementary NAD^+^ replacement strategies fail to functionally protect dystrophin-deficient muscle

**DOI:** 10.1186/s13395-020-00249-y

**Published:** 2020-10-22

**Authors:** David W. Frederick, Alan V. McDougal, Melisa Semenas, Johanna Vappiani, Andrea Nuzzo, John C. Ulrich, J. David Becherer, Frank Preugschat, Eugene L. Stewart, Daniel C. Sévin, H. Fritz Kramer

**Affiliations:** 1 Muscle Metabolism Unit, GlaxoSmithKline R&D, Research Triangle Park, NC, Collegeville, PA USA; 2 Cellzome, GlaxoSmithKline R&D, Heidelberg, Germany; 3 Target Sciences, Computational Biology, GlaxoSmithKline R&D, Collegeville, PA USA; 4 Computational Sciences, Molecular Design, GlaxoSmithKline R&D, Collegeville, PA USA

**Keywords:** MDX, NAD+, CD38, NR, Eccentric, Injury, Metabolomics, Therapeutics

## Abstract

**Background:**

Duchenne muscular dystrophy (DMD) is a progressive muscle wasting disorder stemming from a loss of functional dystrophin. Current therapeutic options for DMD are limited, as small molecule modalities remain largely unable to decrease the incidence or mitigate the consequences of repetitive mechanical insults to the muscle during eccentric contractions (ECCs).

**Methods:**

Using a metabolomics-based approach, we observed distinct and transient molecular phenotypes in muscles of dystrophin-deficient MDX mice subjected to ECCs. Among the most chronically depleted metabolites was nicotinamide adenine dinucleotide (NAD), an essential metabolic cofactor suggested to protect muscle from structural and metabolic degeneration over time. We tested whether the MDX muscle NAD pool can be expanded for therapeutic benefit using two complementary small molecule strategies: provision of a biosynthetic precursor, nicotinamide riboside, or specific inhibition of the NAD-degrading ADP-ribosyl cyclase, CD38.

**Results:**

Administering a novel, potent, and orally available CD38 antagonist to MDX mice successfully reverted a majority of the muscle metabolome toward the wildtype state, with a pronounced impact on intermediates of the pentose phosphate pathway, while supplementing nicotinamide riboside did not significantly affect the molecular phenotype of the muscle. However, neither strategy sustainably increased the bulk tissue NAD pool, lessened muscle damage markers, nor improved maximal hindlimb strength following repeated rounds of eccentric challenge and recovery.

**Conclusions:**

In the absence of dystrophin, eccentric injury contributes to chronic intramuscular NAD depletion with broad pleiotropic effects on the molecular phenotype of the tissue. These molecular consequences can be more effectively overcome by inhibiting the enzymatic activity of CD38 than by supplementing nicotinamide riboside. However, we found no evidence that either small molecule strategy is sufficient to restore muscle contractile function or confer protection from eccentric injury, undermining the modulation of NAD metabolism as a therapeutic approach for DMD.

## Background

Dystrophinopathies are a class of diseases manifesting primarily in skeletal muscle and caused by a variety of mutations in the 2.4 Mb *dystrophin* gene which render the dystrophin protein inactive. Dystrophin is a central force-transducing element of skeletal muscle, connecting the actin cytoskeleton to the extracellular matrix via the dystrophin-glycoprotein complex (DGC). In patients with Duchenne muscular dystrophy (DMD), the absence of functional dystrophin leads to limb muscle weakness, followed by gradual muscle atrophy, cardiomyopathy, and premature death. Dystrophin-deficient muscle is especially susceptible to damage following eccentric contractions, in which the muscle lengthens while generating opposing force, as occurs during the act of sitting, descending stairs, and other activities of daily life [1]. Damaged muscle fibers then undergo repeated cycles of clearance by the immune system and replacement by newly differentiated progenitor cells, in a process that spans weeks. Though progress has been made in addressing the primary defects in *dystrophin* via exon skipping or gene-replacement therapies [2], means of mitigating the effects of eccentric injuries to the muscle of DMD patients have primarily been limited to treatment with palliative anti-inflammatory drugs.

The ability of muscle to harness chemical energy through aerobic and anaerobic respiration is inherently linked to its physical structure. Accordingly, analytical techniques such as NMR and mass spectrometry can detect chemical biomarkers of specific muscular dystrophies, which correlate with the cause and severity of the disease, and can aid in identifying points of therapeutic intervention. For example, it has long been appreciated that the muscle of adult dystrophin-deficient MDX mice contains lower levels of energy-storing and redox-active metabolites, such as phospho-creatine, ATP, and beta-hydroxybutyrate [3], yet high levels of taurine and non-polar amino acids [4]. More recently, distinct metabolomes were also identified in populations of the MDX muscle-resident cells involved in regeneration, including satellite cells and adipose progenitors [5]. Attempts to preserve muscle function by restoring specific metabolic intermediates or co-factors in dystrophic muscle have shown hints of efficacy. For example, supplementing the TCA cycle intermediate, alpha-ketoglutarate (aKG), to the buffer of isolated MDX muscles was reported to improve fatigue resistance ex vivo [6]. In dystrophic mice harboring mutations in the *FKRP* gene, aberrant glycosylation of the DGC component, alpha-dystroglycan, can be partially overcome by supplementing the drinking water with 5% ribitol, the substrate of the mutated enzyme [7]. These studies suggest that specific aspects of the pathophysiology of muscular dystrophies arise from metabolic bottlenecks that may be bypassed using exogenous small molecules.

Nicotinamide adenine dinucleotide (NAD^+^ or NAD) is an essential metabolic co-factor, which has been directly implicated in the maintenance of muscle mass and function during sarcopenia and other dystrophic states [8–11]. NAD has also been found to be depleted in the muscle of MDX mice [10, 12], suggesting potential for therapeutic intervention. At present, two complementary strategies exist for manipulating tissue NAD pools. The first, and most commonly studied strategy, is to enhance NAD production by supplementing biosynthetic precursors, such as nicotinamide riboside (NR) or nicotinamide mononucleotide, to cells with a functional NAD salvage pathway [13]. The second strategy is to inhibit the enzymatic consumption of NAD by several classes of enzymes, including sirtuins, poly-ADP-ribose polymerases (PARPs), and ADP-ribosyl cyclases (ARCs). CD38, the most widely expressed ARC, has been therapeutically targeted for indications ranging from multiple myeloma to neurodegeneration [14]. Our group has previously shown that synthetic small molecule antagonists of CD38 are capable of acutely increasing NAD in muscle and liver tissue [15, 16], and others have reported that chronically dosing one of these thiazoloquinolin(on)es, known in the literature as compound 78c, mitigated structural remodeling and functional decline in the muscle of aged mice [11]. Despite these preliminary findings, the extent to which NAD depletion is a pathologically relevant or therapeutically tractable feature of dystrophinopathies remains unclear.

Recent reports have specifically investigated the potential for NAD supplementation to counteract the pathology of DMD [10, 17]. Here, we have examined this topic further by characterizing the global metabolome of MDX muscle following eccentric challenge and identifying distinct stages of metabolic crisis and repair on the biochemical level. We found evidence that NAD is depleted both acutely and chronically in the muscle of MDX mice with wide-ranging metabolic consequences. We further attempted to reverse the NAD depletion using a novel, highly potent imidazoquinoline inhibitor of CD38 and compared its efficacy to nicotinamide riboside during repeat bouts of eccentric challenge and recovery. Our results may guide the development of NAD-targeting therapeutics for muscle diseases.

## Results

### Dystrophin deficiency alters the muscle NAD metabolome and energy producing pathways

**Fig. 1:**
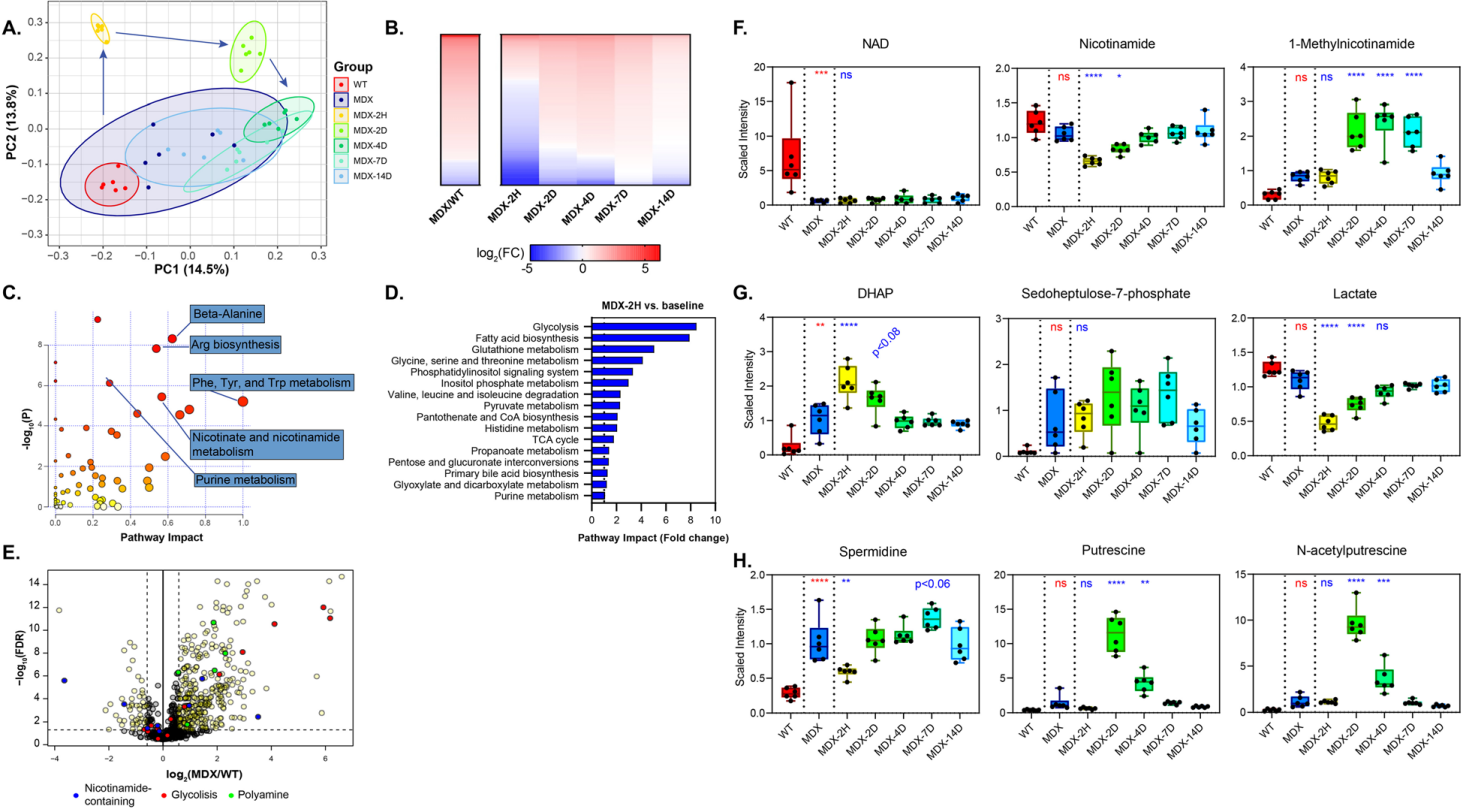
The metabolome of dystrophin-deficient muscle acutely responds to eccentric challenge. **a** Principal component analysis of the global metabolic profiles of MDX gastrocnemius muscles before and after eccentric contractions. Unchallenged WT muscles were included as a negative control. Arrows indicate the early chronological progression of injured groups and ellipses indicate 95% confidence intervals. **b** Heat map of metabolites with significantly different ion abundances in MDX muscle compared to WT (left) and sequential post-injury time points in MDX mice compared to uninjured controls (right). **c** Volcano plot of biochemical pathways significantly altered in MDX muscle at baseline. Significantly altered metabolites were used to calculate pathway impact and highly affected pathways are indicated. **d** Fold change of pathway impact scores during the acute (2 h) injury stage of MDX muscle compared to MDX baseline. **e** Volcano plot of metabolites significantly altered in MDX muscle at baseline. Example components of the NAD metabolome (red), glycolytic intermediates (blue), and polyamine pathway intermediates (green) are indicated. Dashed line indicates the significance threshold of non-adjusted *p* < 0.05. **f** Time course box and whisker plots of selected components of the NAD metabolome, glycolytic intermediates (**g**), and polyamine pathway (**h**) over 14 days post-challenge. *N* = 6 mice per time genotype and time point. Whiskers represent minimum and maximum values. Significance was determined by one-way ANOVA with Tukey’s post hoc test (*p < 0.05, **p < 0.01, ***p < 0.001, ****p < 0.0001; ns, not significant; all *p* values non-adjusted) versus unchallenged WT (red) or MDX (blue) samples

To assess the biochemical adaptations of muscle to dystrophin deficiency, we analyzed the gastrocnemius muscles of MDX mice before and after eccentric challenge using an untargeted metabolic profiling platform. The platform utilized a combination of GC/MS and LC/MS in positive and negative ion mode to identify 762 chemical entities, of which 552 were annotated as either polar metabolites or lipids. Principal component analysis of the metabolic profiles demonstrated a clear clustering of samples by time and genotype (Fig. 1a). Furthermore, the MDX samples showed a well-defined chronological progression following damage. The clustering of samples observed 2 h post-damage showed distinct separation from the other time points, suggesting a period of acute crisis in the muscles. This was the only time at which the majority of significantly altered metabolites appeared depleted when compared to unchallenged controls (Fig. 1b), potentially indicating rapid degradation, release from the tissue, or a synthetic bottleneck. The pattern of the post-injury sample groups from days 2-7 reflected a transition from the acute response to a repair and recovery stage. By post-injury day 14, the samples had a chemical phenotype closely resembling that of the uninjured MDX muscles (Fig. 1a-b).

Comparing the metabolic profiles of MDX mice to wildtype (WT) mice at baseline, we identified several biochemical pathways with a disproportionate impact on the muscle. Among the highest confidence pathways (*p* < 10^ −5 and pathway impact> 0.5) were those relating to the biosynthesis and metabolism of amino acids, such as arginine, phenylalanine, tyrosine, and tryptophan, as well as that of nicotinate and nicotinamide metabolism (Fig. 1c). Comparing the pathway impact scores from the acutely injured (2 h) to uninjured MDX muscle, we were surprised to find glycolysis as the most responsive pathway (Fig. 1d). Additional pathways relating to energy production or storage, including those of fatty acid synthesis, pyruvate metabolism, and the TCA cycle, were also injury responsive.

Upon examining the specific metabolites altered at baseline in the MDX muscle, we found a strikingly lower abundance of NAD than almost any other metabolite (Fig. 1e, f). Consistent with the pathway analysis, several other nicotinamide-containing metabolites and glycolytic intermediates also showed highly variable abundance. Primary NAD deficiency in mouse muscle and cultured myotubes has been shown to restrict glycolytic flux at the level of GAPDH, resulting in a characteristic buildup of intermediates in the pentose phosphate pathway [8, 18]. Consistent with this model, we observed a significant increase in ion counts for glucose-6-phosphate (G6P), dihydroxyacetone phosphate (DHAP), ribose-5-phosphate, and a positive trend in sedoheptulose-7-phosphate (S7P) in MDX muscle (Fig. 1e, g). Additionally, we noted an increase in several poly-cationic species of polyamines, known to be derived from arginine (Fig. 1e, h). Collectively, this pattern indicates a metabolic shift in MDX muscle at a steady state, partially stemming from the loss of the metabolic co-factor, NAD.

The robust response of the global metabolome at 2 h post-eccentric challenge led us to investigate NAD-related metabolites at this and subsequent time points. As far as the platform could resolve, NAD itself did not appear to respond to the challenge (Fig. 1f). However, Nam was acutely depleted by more than one-third after 2 h, presumably restricting any residual activity of the NAD salvage pathway, and gradually returned to baseline after 4 days. Nam homeostasis was further altered by a doubling in the levels of 1-me-Nam in the 2 days following injury, which only normalized after 14 days. Evidence of a further constriction in glycolysis also emerged post-injury: levels of DHAP, immediately upstream of GAPDH, acutely increased in an opposing pattern to that of lactate, a glycolytic end-product (Fig. 1g). The most striking indicator of the repair phase was the appearance of polyamines, including spermine, spermidine, putrescine, and N-acetylputrescine, which were elevated in the days following injury (Fig. 1h and Supplemental Table 1). This class of biomolecules serves as a general marker of cellular proliferation and is required for both myocyte differentiation and alternative macrophage activation [19, 20]. Consistently, in the case of NAD-related metabolites, glycolytic intermediates, and polyamines, eccentric injury amplified the disparities between MDX and WT muscle.

### A novel synthetic CD38 antagonist increases NAD in multiple tissues

**Fig. 2:**
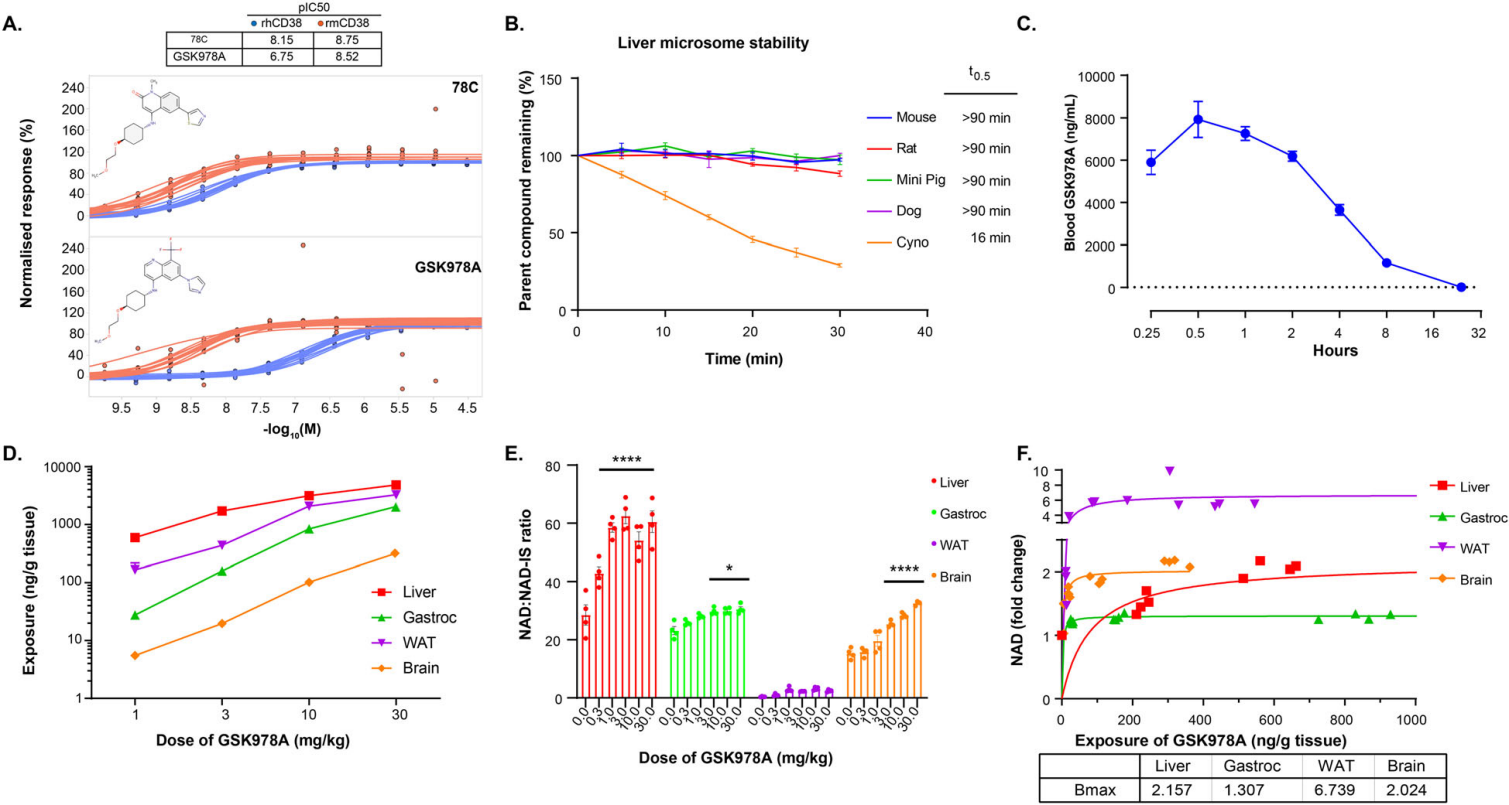
In vivo characterization of a novel synthetic CD38 inhibitor. **a** Inhibition of the base-exchange activity of recombinant human (rh) or mouse (rm) CD38 protein using the established CD38 inhibitor 78C and the novel inhibitor GSK978A. Sigmoidal dose-response curves were fitted to replicate experiments (*n* = 12-15). Molecular structures and calculated pIC50 values are indicated. **b** Stability of 500 nM GSK978A in liver microsomal fractions from selected preclinical species over time. Half-lives (*t*_0.5_) were calculated from linear regressions. *N* = 3 experiments per species. **c** Pharmacokinetics of GSK978A in mouse blood after an oral dose of 10 mg/kg. The dashed line indicates approximate IC50 of 12 ng/mL. *N* = 3 mice. **d** Intratissue compound exposures 2 h after a range of oral doses of GSK978A. *N* = 4 mice per dose. WAT, white adipose tissue. **e** Tissue-specific pharmacodynamics measured as NAD content relative to an internal standard (IS) 2 h after 0-30 mg/kg oral doses of GSK978A. Significance was determined by one-way ANOVA with Tukey’s post hoc test (*p < 0.05, ****p < 0.0001 compared to vehicle controls). f Tissue-specific pharmacodynamics as a function of exposure measured as normalized change in NAD content 2 h after 0-3 mg/kg doses of GSK978A. Lines indicate sigmoidal best-fit regressions approaching the indicated Bmax limits. *N* = 4 mice per dose. Mice were WT obese males for subpanels **c**-**f**. Error bars represent SEM

Our group previously reported a series of novel chemical entities (NCEs), which potently inhibit the constitutive NAD-degrading enzyme, CD38. Related screening efforts yielded the imidazoquinoline dubbed GSK978A, which exhibited tenfold higher potency against mouse recombinant CD38 than the human enzyme (Fig. 2a). This potency is orders of magnitude greater than that of natural products, such as quercetin, and approximates that of 78c, the best-studied synthetic CD38 inhibitor to-date [11, 14]. Yet GSK978A was more soluble and outperformed 78c in a chromosomal stability test of genotoxicity, indicating improved suitability for long-term administration (data not shown). GSK978A was also predicted to have low intrinsic clearance in several small animal preclinical species, including mice and rats, but not larger cynomolgus monkeys (Fig. 2b). As literature suggested a role for CD38 in the prevention of diet-induced obesity [21], the drug metabolism and pharmacokinetic characterization of the quinoline series was originally performed in obese WT mice. To confirm the slow clearance kinetics in vivo, we administered a single intermediate oral dose of 10 mg/kg and found the compound still detectable in the blood after 24 h (Fig. 2c). We next performed a pharmacokinetic analysis of tissues sampled 2 h after oral doses from 1-30 mg/kg. At these doses, the compound was identified within the liver, gastrocnemius, adipose, and brain tissues at exposures that well exceeded the IC50 of ~ 12 ng/mL (Fig. 2d). Accordingly, the NAD content was found to be significantly elevated in the liver, muscle, and brain when normalized to an internal analytical standard (Fig. 2e). Despite high exposure, the NAD recovery from adipose was low and NAD changes were not significant. However, in most tissues, peak NAD elevation of at least 30% was achieved at a dose of 3 mg/kg (Fig. 2f).

**Fig. 3:**
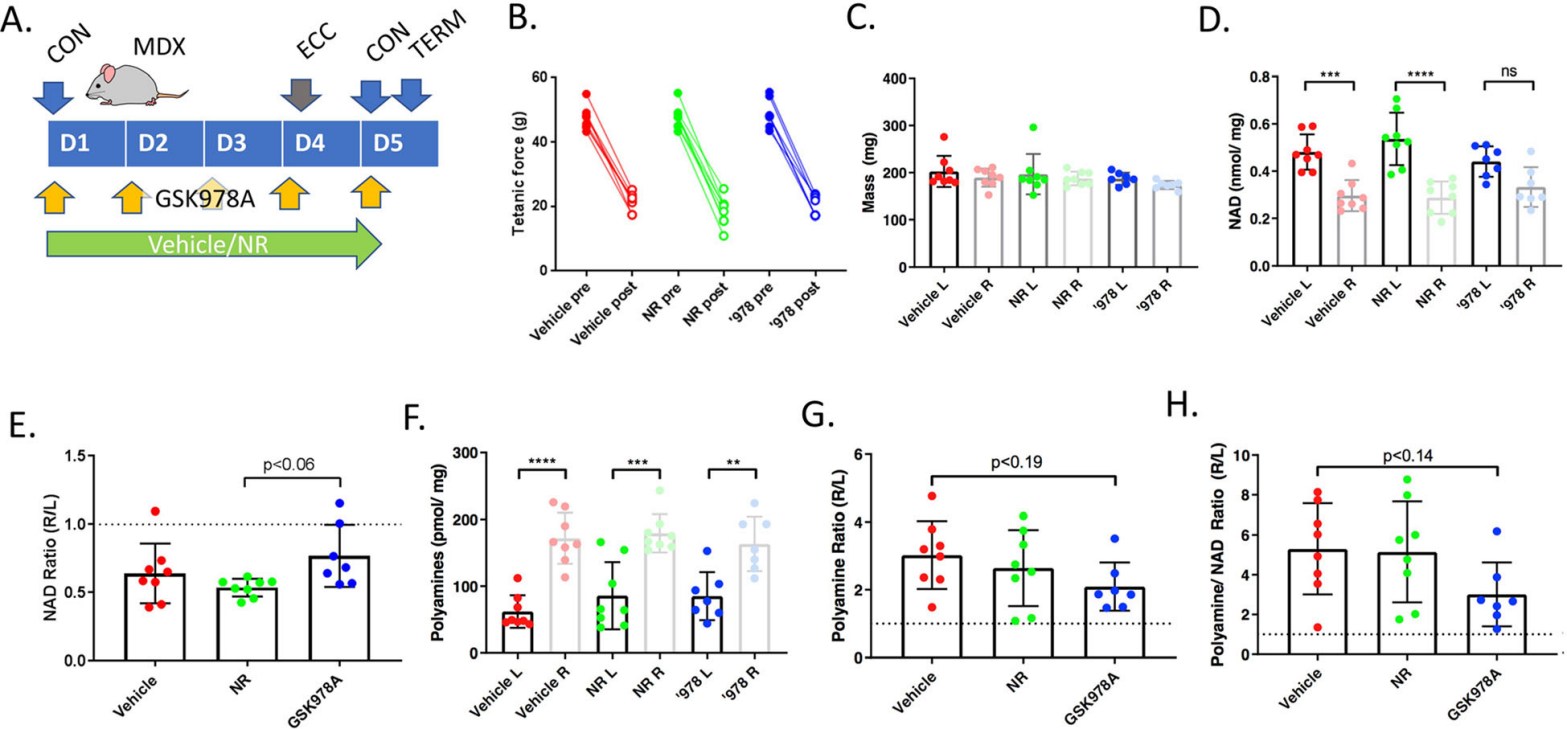
Acute pharmacodynamics of GSK978A and NR in MDX hindlimbs following eccentric challenge. a Schematic of acute study design in MDX mice over 5 days (D1-D5) showing timing of eccentric contractions (ECC), assessments of contractility (CON), termination and tissue harvest (TERM), and compound administration. **b** Comparison of maximal tetanic force produced in the same limb at baseline and following eccentric challenge (ECC). Contractility (CON) was assessed over the 5-day protocol (D1-D5). **c** Masses of gastrocnemius muscles harvested from challenged (right, R) and contralateral (left, L) limbs at the study conclusion. **d** Total and (**e**) relative NAD content of gastrocnemius muscles harvested from challenged and contralateral limbs. **f** Total and (**g**) relative polyamine content of gastrocnemius muscles harvested from challenged and contralateral limbs. **h** Normalization of total polyamine to NAD contents in challenged limbs compared to the contralateral side. *N* = 8 MDX mice per group. Error bars represent SEM. Significance was determined by one-way ANOVA with Tukey’s post hoc test (**p < 0.01, ***p < 0.001, ****p < 0.0001; ns, not significant or *p* value indicated)

To assess the potential pharmacodynamics of GSK978A in an eccentric challenge model, an acute study was performed in MDX mice following 5 days of dosing at 3 mg/kg. Dietary NR, which has been suggested to improve the performance of MDX muscle [10], was included as a comparator. Within 24 h of eccentric challenge, tetanic strength was lessened by > 50% in all treatment groups, despite preservation of mass in the largest affected gastrocnemius muscles (Fig. 3a-c). Interestingly, the challenged muscles also showed NAD depletion compared to the contralateral side, indicating that the muscle NAD pool does acutely respond to lengthening contractions (Fig. 3d). Mice treated with GSK978A, but not NR, showed a trend toward protection from this effect, though it could not be attributed to specific NAD elevation in either limb (Fig. 3e). As a biomarker of muscle repair, total muscle polyamines showed clear elevation in the injured limbs with a trend toward protection by GSK978A, especially when polyamines were normalized to NAD content (Fig. 3f-h). These results suggested that a longer treatment regimen might be necessary to provide functional improvements to MDX mice.

### Chronic NAD repletion does not provide functional protection from repetitive eccentric challenges

**Fig. 4:**
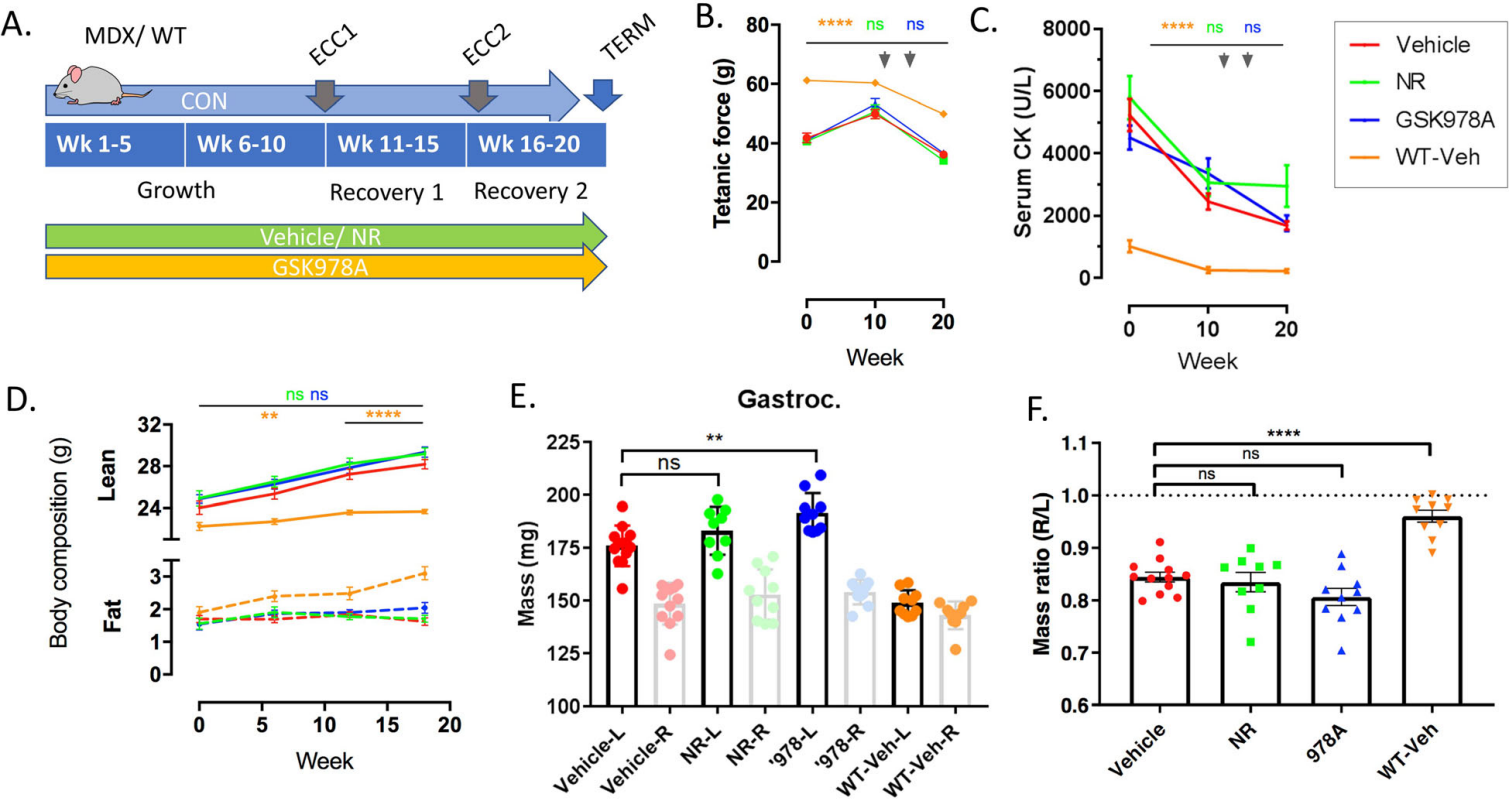
Chronic pharmacodynamics of GSK978A and NR in MDX hindlimbs following eccentric challenge. **a** Schematic of chronic study design in MDX and WT mice over 20 weeks (weeks 1-20) showing timing of eccentric contractions (ECC), assessments of contractility (CON), termination and tissue harvest (TERM), and compound administration. GSK978A was dosed daily and contractility was assessed at varying intervals. **b** Maximal tetanic force production and (**c**) serum creatine kinase activity assessed at baseline, midpoint, and study conclusion. Arrows indicate timing of eccentric challenges. Significance was determined by repeated measure two-way ANOVA with Tukey’s post hoc test relative to MDX vehicle controls (**p < 0.01, ****p < 0.0001; ns, not significant compared to MDX vehicle controls). **d** Body composition assessed by qNMR at baseline, week 8, and week 18 of treatment. **e** Mass and (**f**) mass ratio of injured and contralateral gastrocnemius muscles collected after 20 weeks of treatment. *N* = 9-12 mice per group. Mice are MDX unless otherwise noted. Error bars represent SEM. Significance was determined by one-way ANOVA with Tukey’s post hoc test (****p < 0.001, **p < 0.01; ns, not significant)

We next designed a long-term study with chronic administration of GSK978A or NR to longitudinally assess the physiology of MDX mice during three distinct stages: growth, recovery from an eccentric challenge, and recovery from a repeated challenge (Fig. 4a). We reasoned that this design would model the efficacy requirements of boys diagnosed with DMD. Beginning at 7-9 weeks of age, during a period of rapid growth and peak muscle necrosis [22], MDX mice were randomized by hindlimb contractility and body weight, with a WT group included as a positive control for recovery. Over the course of 20 weeks, hindlimb weakness and increased CK release persisted in the MDX mice compared to WT controls (Fig. 4b-c). The pattern of hindlimb strength and serum creatine kinase (CK) release generally trended downward as MDX animals reached maturity, but remained unchanged in both compound-treated groups, compared to the vehicle-treated controls. MDX mice also accumulated lean mass steadily over the course of the study, reflecting characteristic hypertrophy, in a manner that was treatment-independent (Fig. 4d). At the study conclusion, gastrocnemius muscles from the challenged MDX limbs were found to be ~ 15% less massive than the contralateral side in all treatment groups, reflecting an inability to fully regenerate injured fibers that was not observed in the WT. Surprisingly, contralateral muscles tended to be largest in mice treated with GSK978A (Fig. 4e-f), which may be a consequence of altered gait mechanics to favor the contralateral side, as it was not reflected in total lean mass.

**Fig. 5:**
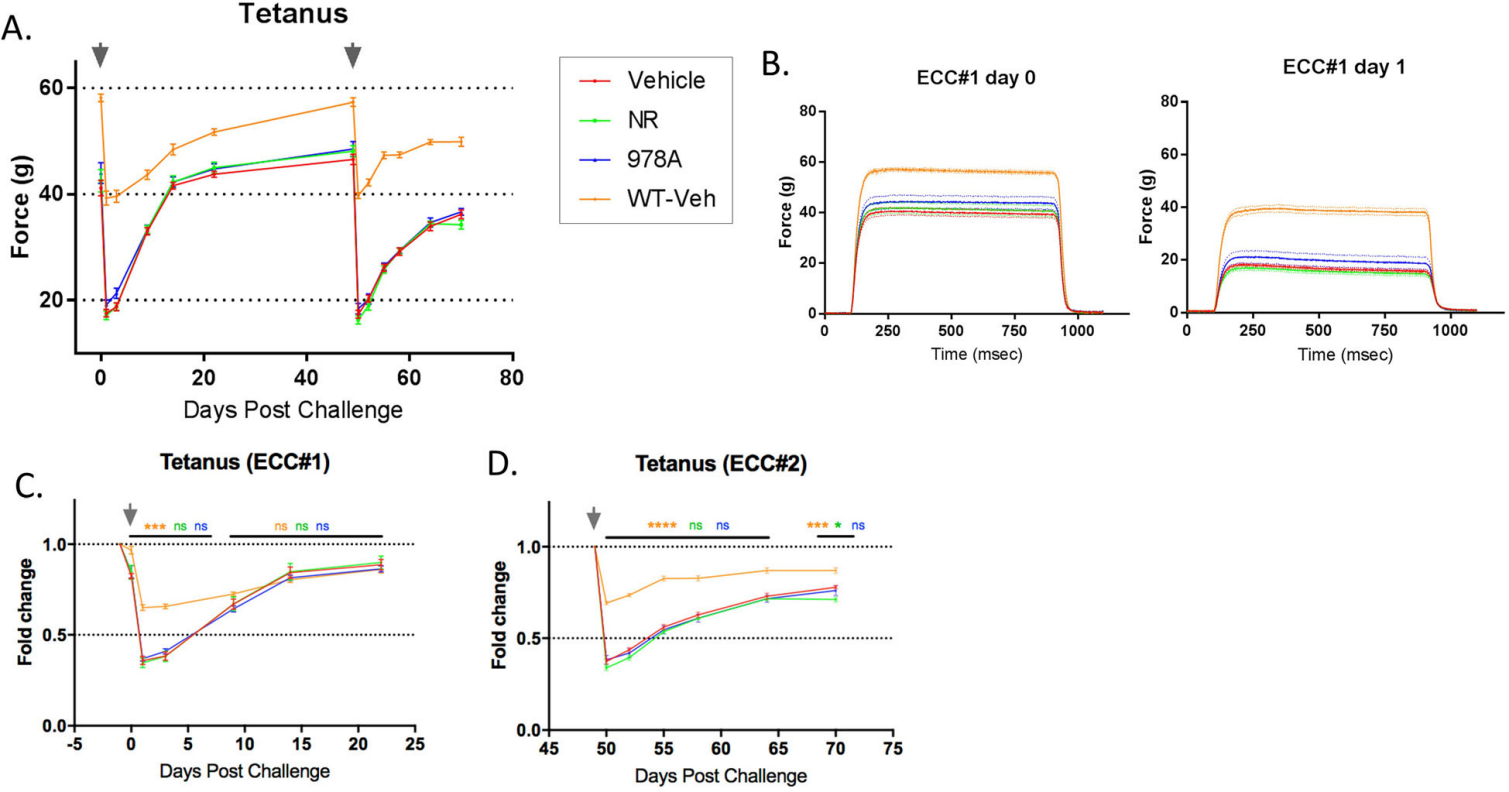
Time course of hindlimb contractile function following consecutive eccentric challenges. **a** Longitudinal tetanic force produced by the hindlimbs beginning at week 10 of treatment and including repeat bouts of eccentric challenge (arrows) and recovery. **b** High-resolution traces of tetani immediately before and 24 h after the first eccentric challenge. **c** Three-week longitudinal hindlimb force production normalized to pre-challenge levels after the first and (**d**) second successive eccentric challenges. *N* = 9-12 mice per group. Mice are MDX unless otherwise noted. Error bars represent SEM. Significance was determined by repeated measure two-way ANOVA with Tukey’s post hoc test (*p < 0.05, ***p < 0.001, ****p < 0.0001; ns, not significant compared to MDX vehicle controls)

Hindlimb strength was also serially assessed following eccentric challenges beginning at week 10 of treatment, to determine whether treated groups were protected from injury or recovered faster. Compared to WT controls, MDX mice showed approximately double the functional deficit within 1 day of eccentric challenge, despite similarly shaped tetani, but neither parameter was affected by GSK978A nor NR (Fig. 5a-b). Furthermore, MDX hindlimbs showed highly similar recovery kinetics between the first and second challenges, while WT controls rebounded faster after the second bout (Fig. 5c-d). The absence of a protective repeated-bout effect in MDX hindlimbs may reflect the fact that adult dystrophin-deficient muscles are preconditioned to such cycles of damage and repair by activities of normal living.

### CD38 inhibition significantly reverts the MDX muscle metabolome to the WT state

**Fig. 6:**
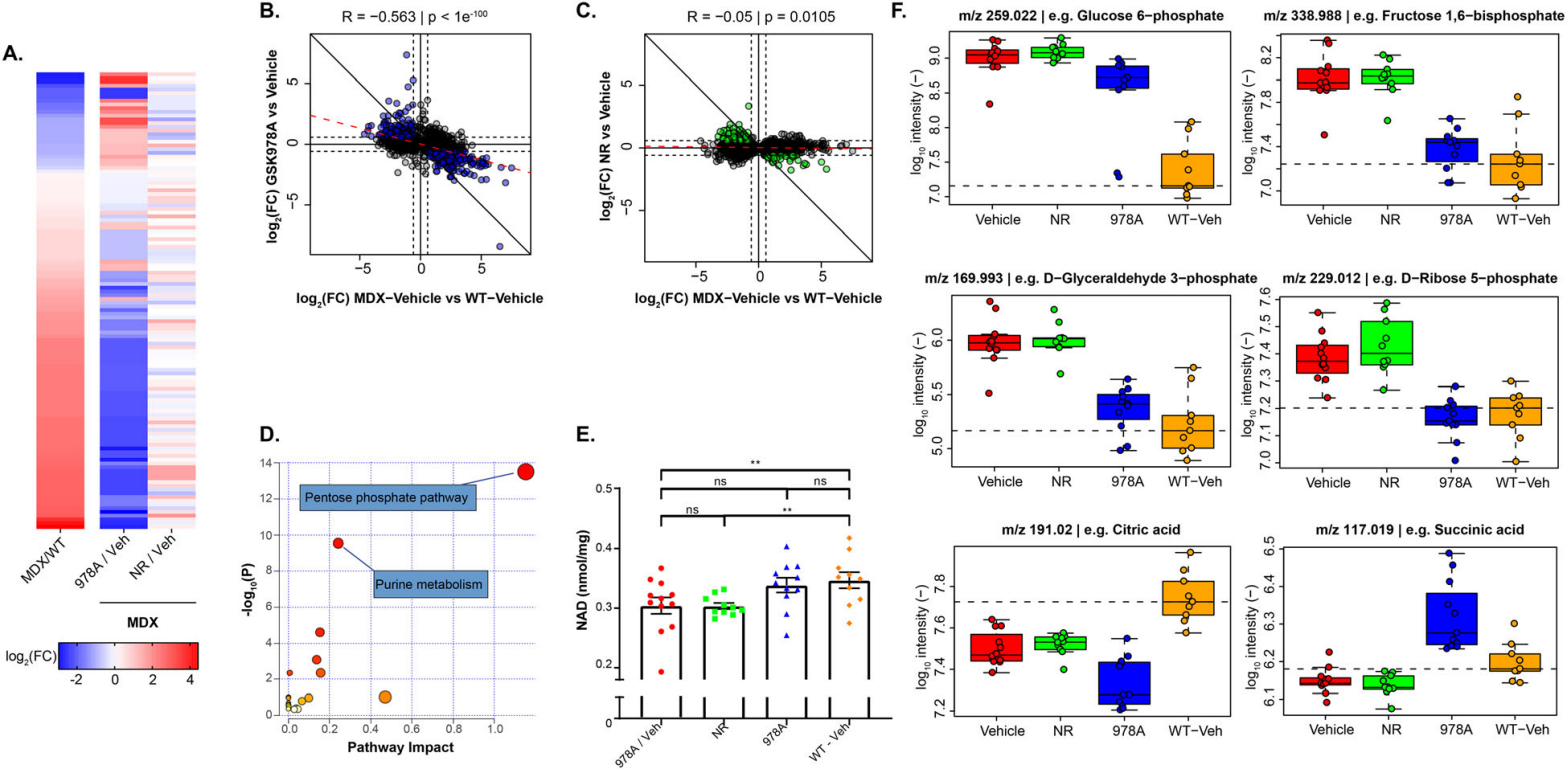
Metabolomic signatures of NAD replacement strategies in MDX muscle. **a** Heatmap of ion counts for polar metabolites reaching significance (adjusted *p* < 0.05) in MDX muscle compared to WT controls (left column) aligned with the same metabolites identified in GSK978A-treated (center column) and NR-treated (right column) muscles after 20 weeks of treatment. **b** Correlation of the changes in ion abundance induced by GSK978A and (**c**) NR supplementation in MDX muscle after 20 weeks compared to changes driven by genotype alone. Colored points indicate metabolite ions significantly altered in at least one of the comparisons and the dashed line indicates the least-squares regression of these points. R indicates the correlation coefficient of the significant (adjusted *p* < 0.05) ions and *p* indicates the significance level of the associated Pearson’s correlation. **d** Volcano plot of biochemical pathways significantly altered in MDX muscle by GSK978A. Significantly altered metabolites were used to calculate pathway impact and highly affected pathways are indicated. **e** Total NAD content of gastrocnemius muscles harvested from contralateral limbs. Significance was determined by one-way ANOVA with Tukey’s post hoc test (**p < 0.01; ns, not significant). **f** Box plots of ion abundances for representative members of glycolysis (G6P, F6BP, G3P), pentose phosphate pathway (R5P), and TCA cycle (CA, SA). *N* = 9-12 mice per group. Whiskers represent minimum and maximum values. Significance was determined by one-way ANOVA with Tukey’s post hoc test (*p* values adjusted for multiple testing)

Given the lack of physiological protection conferred upon MDX muscle by NAD-modulating compounds, we suspected that the treatments were simply ineffective at correcting the basal metabolic imbalance that formed the basis for our rationale. To address this question, we analyzed the uninjured gastrocnemius muscles from the chronically treated mice using a second untargeted metabolomics platform, which utilized LC/MS to provide broader coverage of the negatively charged metabolome, including organic acids. This platform detected 3415 putatively annotated polar metabolites, based on mass. When comparing the metabolites significantly altered between vehicle-treated MDX and WT groups, a reversing pattern emerged in the GSK978A-treated muscles, which was not observed in the NR-treated MDX animals (Fig. 6a). This anti-correlation was confirmed with robust significance (*R* = −0.56, *p* < 1E^ −100) only in the GSK978A treatment group (Fig. 6b-c).

Suspecting that such a dramatic reversion effect may have resulted from restoration of a pleiotropic co-factor, like NAD, we again performed a pathway analysis. Surprisingly, we found the most significant enrichment in only two pathways: purine metabolism and the pentose phosphate pathway (Fig. 6d). Since the untargeted platform was limited in its ability to address the central question of whether positively charged NAD was specifically restored, we instead utilized an enzymatic NAD assay on the same tissue samples and found only statistically non-significant elevations compared to MDX vehicle controls (Fig. 6e). Though the NR-treated MDX muscles still contained significantly less NAD than WT muscle, the intermediate GSK978A group was statistically indistinguishable from vehicle-treated groups of either genotype.

Though sustained NAD elevations were not observed, we found several metabolic reversions consistent with transient restoration of NAD-dependent processes. For example, NAADH, the neutral reduced form of a suspected biomarker of NAD repletion [23] or overload, was detected only in GSK978A-treated muscles (Supplemental Table 1). Importantly, ion counts for components of the proximal glycolytic pathway, including those for glucose-6-phosphate and fructose-1,6,-bisphosphate, were largely normalized by GSK978A, as were the GAPDH substrate, glyceraldehyde-3-phosphate, and the pentose phosphate intermediate, ribose-5-phosphate (Fig. 6f). We also observed discrepant influence on TCA cycle intermediates and a subtle net effect of increased ATP and decreased phosphocreatine in this group (Supplemental Table 1), potentially indicating elevated substrate-level phosphorylation. Collectively, these global metabolomic profiles of dystrophin-deficient muscle highlight a restorative effect of CD38 inhibition on multiple metabolic pathways that is not recapitulated by supplementing a NAD precursor.

## Discussion

Advancements in high-throughput discovery metabolomics have led to improved biomarker detection and novel therapeutic strategies for many diseases, yet determining whether a complex molecular signature is a cause or consequence of pathology is highly context-dependent [24]. Given its central role in maintaining ATP generation via both glycolysis and oxidative phosphorylation, NAD is a co-factor well-positioned to influence muscle mass and performance, and its synthesis and degradation have been suggested to be dysregulated in the absence of dystrophin [10, 12]. Our study confirms that dystrophin-deficient muscle maintains a diminished NAD pool, even in the absence of deliberate physiological challenges. Our observation that NAD can be further depleted within hours of an eccentric challenge suggests that the repetitive nature of these lengthening contractions during activities of normal living may collectively drive chronic NAD depletion in diseased tissue, providing insight into the etiology of DMD. Though the degree to which wildtype dystrophin acutely mitigates, this process remains unresolved, associated therapeutic indications would be limited to less life-threatening conditions, such as exercise recovery or muscular trauma.

The mechanism of acute muscle NAD depletion likely reflects an imbalance in production and consumption fluxes. One model suggests that calcium dysregulation, stemming from microtears in the sarcolemma, leads to a burst of genotoxic reactive oxygen species and hyperactivation of NAD-consuming PARPs [25, 26]. However, cleavage of NAD by PARPs would be expected to liberate nicotinamide, and our data clearly indicate the opposite pattern. Rather, our finding that the methylated waste product, 1-me-Nam, is more abundant in the days following injury, suggests that the removal of nicotinamide equivalents from the cytosol by the enzyme nicotinamide N-methyltransferase (NNMT) may effectively limit the re-synthesis of NAD from nicotinamide via the NAD salvage pathway. The regulation of NNMT activity is still poorly understood [27], and bulk tissue analysis is unable to resolve whether infiltrating cell types are responsible for the effect, but a consistent pattern of increased *NNMT* expression has been previously reported in muscle biopsies from patients with a variety of dystrophic conditions [10]. Furthermore, since 1-me-Nam is prone to urinary secretion, it may be a useful indicator of efficacy for oligonucleotide-based therapeutics, such as those being tested in the *FKRP* mutant model of limb-girdle muscular dystrophy [28]. We also found elevations in MDX muscle of several positive biomarkers previously identified in models of primary muscle NAD depletion [8, 18], including DHAP and S7P. Consistently, the large-scale metabolic imbalance secondary to NAD depletion appeared to be amplified in MDX muscle by a bottleneck in glycolysis. The near-complete reversal of this imbalance by a small molecule CD38 antagonist provided compelling evidence that depletion of one or more of the pathway's cofactors is largely responsible for the distinctive metabolomic fingerprint.

CD38 is a uniquely complex pharmacological target due to an unusual array of enzymatic activities and modes of regulation. Additionally, the ability of the CD38 extracellular domain to function as a cell surface ligand for CD31 represents a signaling mechanism that may be more effectively disrupted with monoclonal antibodies than small molecules and may contribute to the phenotype of CD38 knockout mice [21]. As an enzyme, CD38 can convert not only NAD but also NADP and nicotinic acid into calcium-mobilizing second messengers, such as cADPR and NAADP, in a manner dependent on both membrane topology and local pH [29]. Thus, despite broad exposure, the specific pharmacodynamic effects of GSK978A might vary by cell type. This is especially true when considering NAD synthesis and consumption fluxes, which vary widely between mouse tissues [30]. Indeed, the turnover of NAD in mouse muscle was recently found to be the slowest of any tissue tested [30], and the degradative activity of other enzymes, such as PARPs, may predominate [31]. It is also possible that GSK978A primarily influences the global metabolic profile of muscle via ancillary effects on calcium homeostasis, which is known to be dysregulated in the absence of dystrophin [26], or infiltrating immune cells. Nonetheless, our conclusion that CD38 inhibition did not functionally protect dystrophin-deficient muscle is in line with that of Spaulding et al., who found that long-term administration of the CD38-inhibiting flavonoid, quercetin, failed to protect isolated MDX muscles from contraction-induced injury [32].

Though the muscle exposure of GSK978A was comparable to that of other tissues, the 30% NAD elevation that we observed 2 h after dosing was modest by comparison to the liver, brain, and adipose, which more than doubled NAD content over the same period (Fig. 2). We also failed to detect significant changes in the muscle NAD pool following acute or chronic treatment. This may be an indication of several factors. First, because muscle makes up a large percentage of body mass, muscle-targeting drugs must have high volumes of distribution. Limited solubility or excessive albumin binding could effectively limit the interaction of quinolones, like GSK978A, with their intended target. Second, the accuracy and variability of NAD quantitation is highly dependent on extraction conditions and analytical techniques. Our reliance on multiple mass spectrometry-based and enzymatic assays made it challenging to reproducibly measure subtle shifts in the NAD pool. Third, there may exist a biological upper limit to the steady-state NAD content of muscle, as suggested by earlier transgenic models [33, 34]. The reversibility of the NMN adenyl transferase enzymes may effectively limit the expansion of the NAD pool in a tissue-specific manner. Lastly, it is possible that CD38 is not a major consumer of NAD in muscle, or that the enzyme expression is downregulated during pathology. Such transcriptional compensation has been observed previously in DMD muscle [10]. Nonetheless, a global assessment of the treated tissues was largely consistent with a transient or compartmentalized restoration of NAD-dependent pathways. This restoration did not manifest in the form of nicotinamide-containing metabolites, as predicted, but rather in a more stable impact on pentose phosphate pathway intermediates. The observed impact on purine metabolism is likely to be directly linked via normalization of ribose-5-phosphate, the pentose phosphate-derived nucleotide precursor (Fig. 6d, f). Interestingly, purine metabolism was identified in our initial characterization of MDX muscle, but was not implicated in the acute response to eccentric injury (Fig. 1c, d). Thus, GSK978A may be more effective at restoring chronic metabolic imbalances, rather than buffering acute challenges.

A central finding of our work is that specific antagonism of CD38 is a more effective strategy than NR supplementation for restoring the metabolic imbalance of MDX muscle. The low micromolar IC50 of GSK978A achieves muscle NAD elevation similar to that of natural products, such as NR [35], at less than 1% of the effective dose. The relative inability of NR to affect the MDX muscle metabolome likely stems from its poor bioavailability and short (< 3 min) half-life in the blood [8, 30], which is consistent with the absence of pharmacodynamics observed in several clinical trials [36–38]. However, both NAD-modulating strategies employed in our study failed to improve muscle function. We were largely unable to reproduce the results of the Auwerx group, who observed a significant reduction of plasma creatine kinase and a nearly 50% protection from eccentric challenge in the same strain of MDX mice treated with NR for only 12 weeks [10]. The discrepancies may derive from the fact that Ryu et al. assessed hindlimb torque around the knee joint instead of the ankle, and administered an NR-triflate salt to mice, instead of the NR chloride salt used in all neutraceutical formulations. Nonetheless, our finding that neither GSK978A nor NR had any effect on the performance of MDX muscle over time raises the question of whether biochemical imbalance is pathologically relevant in the absence of a central structural component like dystrophin. The favorable pharmacokinetics and brain penetrance of GSK978A suggest that the compound may have better efficacy in certain neurodegenerative disorders, which feature NAD depletion, such as Cockayne syndrome or xeroderma pigmentosa [39]. These and other indications for small molecule NAD-modulators warrant further investigation.

## Conclusion

In summary, MDX mice exhibit a chronic NAD deficit with broad effects on the biochemical phenotype of the hindlimb muscle. The distinct global metabolome of dystrophin-deficient muscle becomes acutely altered by eccentric injury and can be partly restored by inhibition of CD38, though this intervention does not confer protection against future injury. While primary NAD deficiency may suffice to drive both muscle weakness and a transcriptional profile resembling dystrophy over time, our current data strongly suggest that the characteristic muscle weakness of MDX mice cannot be overcome by NAD replacement strategies alone and that such strategies would be unlikely to benefit patients with DMD.

## Methods

### Animal care and use

Male C57BL/10ScSn-Dmd<mdx>/J (MDX) and C57BL/10ScSn/J (WT) aged 7-9 weeks were individually housed with *ad libitum* access to regular chow and water during a 12: 12 h light: dark cycle under controlled temperature and humidity. Pharmacokinetic studies were performed in 5-month old C57BL6 mice fed a high fat diet (Research Diets D12492). GSK978A was custom synthesized and dissolved at 0.3 mg/mL in vehicle containing 0.5% hydroxpropyl methyl cellulose and 0.1% polysorbate 80, pH 4. Ten milliliters per kilogram was administered daily in the morning by oral gavage. NR chloride was custom synthesized and dissolved in the drinking water at 12 mM, sterile filtered, and administered *ad libitum* in light protected bottles, as described [8, 40]. All compounds were reformulated weekly. Body composition was monitored by quantitative NMR spectroscopy. All studies were conducted in accordance with the GSK Policy on the Care, Welfare and Treatment of Laboratory Animals and were reviewed the Institutional Animal Care and Use Committee either at GSK or by the ethical review process at the institution where the work was performed.

### Compound screening

NCEs were tested for inhibition of CD38 transglycosidation or base exchange activity by colorimetric assay based on a published method [41] using recombinant mouse CD38 soluble domain protein purified from *Pichia pastoris*. Briefly, 0.5 nM enzyme was incubated in buffer containing 50 mM HEPES, pH 7.4, 1 mM CHAPS, 2 mM EDTA, 250 μM isonicotinaldehyde 2-pyridinylhydrazone, 100 μM NAD, 1% DMSO, and 1-10,000 nM NCEs while absorbance was monitored at 405 nm. Inhibitor potency was calculated with the following equation: *y* = *A*+((*B*-*A*)/(1 + (10^*x*/10^*C*)^*D*)), where *A* is the enzyme-free response, *B* is the inhibitor-free response, *C* is the log (IC50), and *D* is the hill slope.

### Global metabolomics following eccentric challenge (external platform)

Samples were prepared using the automated MicroLab STAR system (Hamilton Company, Franklin MA). Recovery standards were added prior to the extraction process for quality control purposes. Samples were lysed in ice-cold methanol and the resulting extract was divided into four fractions: one each for analysis by reversed-phase UPLC-MS/MS with positive and negative ion mode electrospray ionization, one for normal-phase UPLC-MS/MS platform, and one for analysis by GC-MS. Samples were centrifuged at 13,000×*g* for 10 min and supernatants were dried under nitrogen. The MS system was a Thermo Scientific Q-Exactive high resolution/accurate mass orbitrap mass spectrometer operated at 35,000 mass resolution which was interfaced with a heated electrospray ionization (HESI-II) source. Dried sample extracts were reconstituted in solvents amenable to their respective method. One aliquot was analyzed using acidic positive ion optimized conditions and another using basic negative ion optimized conditions in two independent injections using separate dedicated columns (Waters UPLC BEH C18-2.1 × 100 mm, 1.7 μm). The extracts reconstituted in acidic conditions were gradient eluted using water and methanol containing 0.1% formic acid, while the basic extracts, which also used water/methanol, contained 6.5 mM ammonium bicarbonate. A third aliquot was analyzed via negative ionization following elution from a HILIC column (Waters UPLC BEH Amide 2.1 × 150 mm, 1.7 μm) using a gradient consisting of water and acetonitrile with 10 mM ammonium formate. The MS analyses alternated between MS and data-dependent MS^2^ scans using dynamic exclusion, and the scan range was from 80-1000 m/z. The samples designated for GC-MS analysis were derivatized under nitrogen using bistrimethyl-silyl-trifluoroacetamide (BSTFA). The GC column was a 20 m × 0.18 mm ID, with 5% phenyl; 95% dimethylsilicone phase. Samples were analyzed on a Thermo-Finnigan Trace DSQ fast-scanning single-quadrupole mass spectrometer using electron impact ionization at unit mass resolution. Raw data was extracted, peak-identified, and quality control processed using Metabolon’s hardware and software. Peaks were quantified using area-under-the-curve. Compounds were identified by comparison to library entries of purified standards or recurrent unknown entities. Proprietary visualization software was used to confirm the consistency of peak identification among the samples.

### Global metabolomics following chronic interventions (internal platform)

Polar metabolites were extracted from frozen tissues following lysis in a fivefold excess of ice cold 70% ethanol using a bead homogenizer. Tissue lysates were further diluted 1:20 in 70% ethanol, incubated at 75 °C for 3 min, and centrifuged at 13,000×*g* for 10 min. Supernatants were lyophilized, resuspended in 0.1 mL water, and subjected to flow injection mass spectrometry. Non-targeted mass spectrometry of polar metabolites was performed as described [42]. Briefly, Q-exactive Plus (Thermo Scientific) in profile mode with scan range 50-1000 m/z was calibrated according to manufacturer protocols. Resolution was set to 70,000 at 200 m/z with automatic gain control target of 3E6 ions, 3.0 kV spray voltage, 120 ms maximum injection time, and 60 s acquisition time. Samples were injected in a randomized sequence and analyzed in negative ion mode using a mobile phase consisting of 60% isopropanol, 40% water, 1 mM NH_4_F, 10 nM taurocholic acid, 20 nM homotaurine. Quality control was performed before each batch of ten runs using a standard solution of 16 organic acids. Peak detection and global alignment of all scans was performed using a custom metabolomics data processing pipeline. Detected ion m/z values and isotope distributions were matched against the human metabolome database [43] assuming [M-H] and [M-2H] species and at most two ^13^C/^12^C exchanges to tentatively annotate metabolites, with the method-inherent limitation of being unable to distinguish between isomers.

### Biochemical pathway analysis

Pathway analysis was performed on metabolites reaching an adjusted significance threshold of *p* < 0.05 for a given comparison using the MetaboAnalyst 4.0. platform [44] and referencing the current KEGG pathway library for mouse. Over-representation analysis was performed using Fisher’s exact test and pathway topology analysis was performed using relative-betweeness centrality.

### Tissue pharmacokinetics and pharmacodynamics

To determine the stability of new chemical entities in vitro, cryopreserved liver microsomes from several species (Sekisui Zenotech, Japan) were thawed and diluted to 0.9 mg/mL in 50 mM phosphate buffer, pH 7.4. NCEs in DMSO were added at 0.5 μM to the microsome suspension and pre-incubated for 5 min at 37 °C in a standard cell culture incubator with shaking at 80 RPM. Clearance reactions were started by the addition of 2 mM NADPH and 5 mM MgCl_2_ cofactors, then 100 μL of microsome suspension was removed from the reaction at designated time points and mixed with 200 μL ice-cold stop solution containing 80:20 methanol: acetonitrile containing 1% acetic acid. Microsome extracts were centrifuged at 10,000×*g* for 15 min and supernatants were subjected to LC-MS/MS analysis (below). Metabolic stability expressed as a percentage of the parent compound remaining over time was determined from the peak area ratios in order to calculate the turnover rate constant, *k*, by linear regression and half-life according to the equation *t*_0.5_ = ln(2)/*k*. For assessing NCE distribution and pharmacodynamics in vivo, 10 μL of blood was harvested from the mouse tail vein, mixed with 50 μL of water and 40 μL of acetonitrile. Samples were sonicated for 5 min, vortexed for 5 min, and centrifuged at 2000×*g* for 20 min. Supernatants were diluted 1:5 in water and subjected to LC-MS/MS analysis. Tissues were bead homogenized for 2× 1 min in a fourfold excess of ice-cold 80% acetonitrile and centrifuged at 13,000×*g* for 20 min. Supernatants were diluted 1:10 in water and subjected to LC-MS/MS analysis. As an internal standard, 1.5 μmol of ^18^O-NAD was spiked into the tissue matrix. LC-MS/MS was performed on an Agilent 1290 Infinity system using a mobile phase of methanol containing 0.1% formic acid and a Varian Polaris amide-C18 column coupled to a Sciex API 4000 mass spectrometer. NAD peaks were normalized to the internal standard and drug concentrations were determined using a standard curve generated in the tissue matrix.

### Hindlimb eccentric challenge and longitudinal contractility

Mice were anesthetized using isoflurane (3%/L O_2_) and placed on a warming pad with their right hind limbs restrained at the knee and foot affixed to a force transducer with motor-arm (Aurora Scientific Instruments, Aurora, ON). Platinum sub-dermal electrodes were inserted dorsally and ventrally to the femur to apply electrical field stimulation (2.5 mA at 25 V) to the sciatic nerve and trigger contraction of the plantarflexor muscles of the lower limb. Muscles were stimulated isometrically at a single twitch (200 μs pulse) and tetanic (150 Hz at 200 μs pulse for 0.8 s) frequencies to assess longitudinal force production over the course of the study. Eccentric injury was induced by subjecting hindlimbs to a series of 40 lengthening contractile stimuli, consisting of a sub-tetanic stimulation of 100 Hz at 200 μs pulse for 0.4 s, while the motorized footplate applied an eccentric rotational torque. Animals were returned to holding enclosures and isometric titanic force was monitored to assess force deficit and recovery.

### Acute pharmacodynamics following eccentric challenge

For acute eccentric challenge studies, male C57BL/10ScSn-Dmd<mdx>/J aged 24-26 weeks were individually housed and treated for 5 days, as above. Baseline body weight and contractility were assessed 1 week before the start of dosing and used for group randomization. On day four of treatment, right hindlimbs were subjected to the eccentric damage protocol 15 min after oral dosing. On day five, oral compounds were dosed 15 min before contractility measurement and 60 min before sacrifice. Muscles from both limbs were snap frozen and stored at −80 °C before analysis.

### Creatine kinase measurement

Mice were anesthetized using 3% isoflurane and venous whole blood was collected in a microcapillary from the retro-orbital sinus. Blood was allowed to clot at room temperature for 30 min, then centrifuged at 10,000×*g* for 5 min. The resulting serum samples were diluted 1:3 in water and subjected to automated enzymatic assay (Beckman Coulter, Brea CA).

### NAD and polyamine measurement

NAD was extracted from frozen muscles and measured by enzymatic cycling assay, as described [34]. Briefly, 50 mg of muscle was extracted in 0.5 mL 0.6 M perchloric acid and diluted 1:100 in 100 mM phosphate buffer, pH 8. Samples and NAD standards were further diluted 1:20 in a cycling mix containing 0.1% BSA, 2% ethanol, 100 μg/ml alcohol dehydrogenase, 10 μg/ml diaphorase, 20 μm resazurin, and 10 μm flavin mononucleotide in 100 mm phosphate buffer. Enzymatic cycling at room temperature produced resorufin, the fluorescence of which was monitored over time at ex/em 544/590 nm. Muscle total polyamines were measured using a fluorometric total polyamine assay kit (K475-100, Biovision) according to the manufacturer protocol. Briefly, frozen muscles were ground under liquid nitrogen and a 100 mg portion was further dounce homogenized in 0.5 mL of ice-cold homogenization buffer. Lysates were centrifuged at 5000×*g* for 5 min and supernatants were further filtered through 10 kD molecular weight cutoff spin columns. Extracts were assayed by fluorometric enzymatic assay and compared to a standard curve.

### Statistics

Data were compiled and analyzed using Microsoft Excel and graphed using Graphpad Prism. Statistical tests (Student’s 2-tailed *t* test, one-way ANOVA, repeated measure two-way ANOVA, Tukey’s post hoc test, and least-square correlation analysis) were calculated using Graphpad Prism with a significance threshold of *p* < 0.05, as indicated. For metabolomics data, *p* values were adjusted for multiple hypothesis testing using either Benjamini’s and Hochberg’s method [45] (external platform) or Storey’s and Tibshirani’s method [46] (internal platform data), and principal component analysis was performed on the first two of ten components using custom R scripts.

## Supplementary information

**Additional file 1.** Frederick et al Supplemental Table 1.

## Data Availability

The metabolomics datasets analyzed in the current study are available in the online version of the article. The datasets analyzed in the current study are available from the corresponding author upon request.

## Abbreviations

NCE: Novel chemical entity
TCA: Tricarboxylic acid
DHAP: Dihydroxyacetone phosphate
ECCs: Eccentric contractions
NAD+ or NAD: Nicotinamide adenine dinucleotide
NADP: Nicotinamide adenine dinucleotide phosphate
NAADH: Nicotinic acid adenine dinucleotide (reduced)
NAADP: Nicotinic acid adenine dinucleotide phosphate
DGC: Dystroglycan complex
PARP: Poly-ADP-ribose polymerase
ARC: ADP-ribosyl cyclase
aKG: Alpha-ketoglutarate
NR: Nicotinamide riboside
NNMT: Nicotinamide N-methyl transferase
Nam: Nicotinamide
S7P: Seduheptulose-7-phosphate
F6BP: Fructose-1,6-bisphosphate
CK: Creatine kinase
